# Household fuel use and its association with potential respiratory pathogens among healthy mothers and children in Ethiopia

**DOI:** 10.1371/journal.pone.0277348

**Published:** 2022-11-10

**Authors:** Mulugeta Tamire, Adamu Addissie, Solomon Gizaw, Tamrat Abebe, Shadi Geravandi, Staffan Nilsson, Lucia Gonzales-Siles, Rickard Nordén, Rune Andersson, Susann Skovbjerg

**Affiliations:** 1 Department of Preventive Medicine, School of Public Health, Addis Ababa University, Addis Ababa, Ethiopia; 2 Department of Occupational and Environmental Medicine, Institute of Medicine, Sahlgrenska Academy, University of Gothenburg, Gothenburg, Sweden; 3 Tikur Anbessa Specialized Hospital, College of Health Sciences, Addis Ababa University, Addis Ababa, Ethiopia; 4 Department of Microbiology, Immunology and Parasitology, School of Medicine, College of Health Sciences, Addis Ababa University, Addis Ababa, Ethiopia; 5 Department of Infectious Diseases, Institute of Biomedicine, Sahlgrenska Academy, University of Gothenburg, Gothenburg, Sweden; 6 Department of Laboratory Medicine, Institute of Biomedicine, Sahlgrenska Academy, University of Gothenburg, Gothenburg, Sweden; 7 Department of Clinical Microbiology, Sahlgrenska University Hospital, Region Västra Götaland, Gothenburg, Sweden; Lagos State University, NIGERIA

## Abstract

**Background:**

Over 90% of Ethiopians still rely on solid fuels for cooking food. The pollution from the burning process causes adverse respiratory outcomes including respiratory infections. This study aimed to assess the association of the pollution with nasopharyngeal occurrence of potential pathogens.

**Methods:**

We conducted a comparative cross-sectional study in urban and rural settings in Ethiopia in 2016. Questionnaire-based data were collected from 168 mothers and 175 children aged below two years. Multiplex real-time PCR assays were performed on nasopharyngeal secretions for detection of bacteria and viruses and for the identification of pneumococcal serotypes/groups.

**Results:**

High rates of bacteria and viruses in the nasopharynx were detected by PCR among both the children and the mothers. Among the detected viruses, enterovirus was more commonly detected among rural children than among children from urban areas. *Streptococcus pneumoniae* and *Haemophilus influenzae* were both more prevalent among children and mothers from rural areas compared with urban groups and among those using solid fuels compared with cleaner fuel users. Children from rural households using solid fuels and children whose mothers had educational status below high school had four times higher odds for detection of *S*. *pneumoniae* compared with those households using cleaner energy or those children having mothers with a higher educational status, respectively. One or more serotype/serogroup was identified in about 40% of the samples that were positive for pneumococci. Out of all identified serotypes/serogroups, 43% in the children and 45% in the mothers belonged to PCV13, indicating the larger majority of detected pneumococci being non-PCV13 serotypes.

**Conclusion:**

This study presented a high carriage rate of *S*. *pneumoniae* and *H*. *influenzae* among both children and their mothers, especially in rural areas and among solid fuel users. Thus, interventions should target cleaner energy sources to the public and promote maternal education.

## Background

More than three quarters of households in African and Asian countries still rely on solid fuels, such as wood, dung, charcoal or crop residues, to cook food and heat their houses. Thus, the total number of global populations using solid fuels remains around three billion [1,2]. Ethiopia, as a sub-Saharan African country, has one of the highest rates of solid fuel use as the main energy source at about 90% and nearly 100% in the rural settings [1].

Studies from low- and middle-income countries have shown that such a burning process and the related household air pollution causes several adverse respiratory outcomes. These include impaired lung growth, chronic obstructive pulmonary disease (COPD), lung and nasopharyngeal cancers, tuberculosis and diseases of the eye in adult women, also low birth weight and, of particular concern, acute lower respiratory infections in their small children caused by spending most of their time near the domestic hearth [3,4].

Previous studies have indicated an association between exposure to household air pollution due to solid fuel use and open fire burning and nasopharyngeal carriage of potential pathogens such as *Streptococcus pneumoniae* and *Haemophilus influenzae* among children [5–7] and mothers [5]. A recent prospective study also showed a significant association between PM_2.5_ exposure and risk of *S*. *pneumoniae* carriage in Malawian infants [8]. *S*. *pneumoniae* (the pneumococcus) is a major bacterial pathogen causing respiratory tract infections, with 515,000 deaths among children globally in 2015 [9]. The morbidity and mortality related to pneumonia of children under-five in Ethiopia is still high [10,11], despite the introduction of 13-valent pneumococcal conjugate vaccine (PCV13) in the infant immunisation program in 2011 [12].

Prior studies in Ethiopia demonstrated an association between household air pollution and acute respiratory infections among children indicating the emissions from the use of the solid fuels increase the risk of morbidity and mortality among children in the country [13–16]. We have also previously found a higher occurrence of respiratory symptoms and reduced lung function among Ethiopian women using solid fuels compared with those using cleaner energy [17]. However, there have been no studies, to our knowledge, conducted in Ethiopia to assess the association between household air pollution due to solid fuel use and the nasopharyngeal occurrence of potential pathogens among children or adults related to the type of residence and fuel used. The findings of the study report information, which is relevant for the policy makers and programmers in the area and this could also serve as baseline data for future studies.

## Methods and materials

### Study design and area

This comparative cross-sectional study was carried out from July to August in 2016 in urban and rural settings of Ethiopia: Addis Ababa, the capital city situated in the central part of Ethiopia and the rural area of Butajira in the Gurage zone of the Southern Nations and Nationalities and Peoples Region (SNNPR), approximately 136 km south of Addis Ababa. One urban and nine rural kebeles (the lowest administrative level) of the area have been the site of the Demographic Surveillance System (DSS) of Addis Ababa University Rural Health Program, since 1987 [18]. Solid fuel is the main source of energy in the area for heating and cooking on open fires, mostly by using traditional stoves though improved cook stoves made locally also in use [17]. According to the Ethiopian Demographic and Health survey 2016, the coverage of the general infant vaccination program in SNNPR and Addis Ababa was 47% and 89%, respectively. Thus, at the time of the study, approximately 60% in Butajira and 95% from Addis Ababa were vaccinated with PCV13 and *Haemophilus influenzae* type b vaccine, which were administered simultaneously at the age of 6, 10 and 14 weeks [19].

### Populations and sampling

The study population of mothers and their children were part of another published study, involving 545 mothers aged 19–49 years, included during a six months period in 2016 for assessment of respiratory symptoms and lung function in relation to household fuel use [17]. Of these, 168 mothers (85 from urban setting and 83 from rural) of reproductive age, as well as 175 children (87 from urban and 88 from rural) aged below two years, were included in this study. In 164 cases, the child was the son or daughter of an included mother. In Addis Ababa, mothers and children visiting the health center for vaccination were selected systematically, whereas in Butajira participants were recruited from villages and were appointed to come to the health center as most child vaccination was given in the villages by health extension workers and not at the health centers.

### Questionnaire

Face-to-face interviews were conducted using a structured questionnaire prepared in English, translated to Amharic (national language) with back translation to check for consistency. The questionnaire contained socio-demographic characteristics and questions regarding cooking conditions such as type of fuels, housing and ventilation [17]. The types of fuel the households used were categorized into solid fuel (wood, charcoal, dung or crop wastes) and cleaner energy (liquefied petroleum gas or electricity).

### Collection of specimen and culture of pneumococci

Trained nurses obtained nasopharyngeal secretions from all participating mothers and children by inserting a flocked swab into the nostril and rotating it over the nasopharyngeal surface, before being kept in 1mL of Liquid Amies medium (ESwab^™^, Copan Diagnostics Inc., Murrieta, CA). The samples were transported in ice-boxes to the Bacteriological laboratory at Tikur Anbessa Specialized Hospital, Addis Ababa University, Addis Ababa, and cultured the same day using defibrinated sheep blood agar with addition of gentamicin. The agar plates were incubated for 2 days in 34–36°C, at least the first day in a candle-jar and checked after each day. *S*. *pneumoniae* was identified by colony morphology and optochin sensitivity (≥14 mm). The remaining part of the samples was immediately stored frozen at -80°C until transported to the Department of Infectious Diseases, University of Gothenburg, Gothenburg, Sweden for molecular analysis (see below).

### Bacterial and viral nucleic acid detection

Total DNA was extracted from 200 μL of the nasopharynx secretion sample with a MagNA Pure LC instrument (Roche Diagnostics, Mannheim, Germany) using the Total Nucleic acid Isolation kit (Roche Diagnostic). The DNA was eluted in 100 μL elution buffer and stored at -20°C until further analysis. Detection of fifteen different virus (adenovirus, bocavirus, coronavirus 229E, HKU1, NL63 and OC43, enterovirus, influenza A and B, metapneumovirus, parainfluenza 1–3, rhinovirus and respiratory syncytial virus (RSV)) and of five bacterial species (*S*. *pneumoniae*, *H*. *influenzae*, *Bordetella pertussis*, *Chlamydia pneumoniae* and *Mycoplasma pneumoniae*) was performed as previously described (20). A sample was considered positive if the cycle threshold (Ct)-value was lower than 35, while Ct<30 denoted high amounts of nucleic acids. *S*. *pneumoniae* was identified by either detection of the pneumococcal autolysin *lytA* gene (this assay) or the pneumococcal capsule *cpsA* gene (detected in the serotyping assay, see below) [20,21].

### Serotyping of *S*. *pneumoniae*

A multiplex real-time PCR capable of detecting 40 different serotypes or serogroups in addition to the *cpsA* gene was performed according to Gonzales-Siles et al [21]. The protocol targets the 13 serotypes included in the PVC13, however, it cannot differentiate between the 6A, B, C and D, 7A and F nor 9A and V serotypes. Instead, these are denoted as serogroups 6ABCD, 7AF and 9AV.

### Statistical analysis

Data entry and cleaning using statistical software for epidemiology EPI-Info version 3.5.4 (CDC) were made and exported to IBM SPSS statistics version 24 for analysis. After visualizing the general features of the data, descriptive statistics such as mean and standard deviation for continuous variables and frequency and percentage for categorical variables were determined separately for urban and rural participants. We used Chi-square test of independence to determine whether or not a statistically significant relationship existed between pathogen occurrence in relation to the place of residence and fuel type used. We also used Fisher exact test for cells with an expected count less than five so as not to violate assumptions of the Pearson Chi-square test. One-way-Anova and Tukey’s multiple tests using GraphPad Prism 8.4.2 were used to relate levels of detected bacterial nucleic acids as determined by cycle threshold (Ct) value to place of residence and fuel type. Finally, we applied logistic regression to determine the distribution of the study subjects by independent variable of interest and to see crude associations. Multivariable logistic regression analysis with adjusted odds ratio was used to evaluate relative effects of solid fuel use by including those variables with *p*-value below 0.25 in the bivariate analysis. For all tests, 95% confidence interval was used and the *p*-value was set to <0.05 to determine significance. Fuel users were re-categorized as rural solid fuel users, urban solid fuel users and cleaner energy users to exclude place of residence from the regression model for its multicollinearity with fuel type because all rural households used solid fuel.

### Ethical approval

Ethical approvals were obtained from the Institutional Review Board of the College of Health Sciences of Addis Ababa University, the National Research Ethics Review Committee (NRERC, 3.10/168/2016), Ministry of Science and Technology, Ethiopia and the Regional Ethics Committee in Gothenburg, Sweden (D-nr 115–17). Permissions to conduct the research were received from respective organizations at both settings. All mothers gave informed consent; confidentiality and anonymity were kept throughout the study.

## Results

### Socio-demographic characteristics of the participants

Table 1 shows the basic characteristics of the study participants. The mothers in the urban area were on average 28 years of age compared with 34 years in the rural area. The mean ages of the urban and rural children were 6 and 13 months, respectively. By education, nearly half (48%) of the rural mothers had not attended school at all while 60% in the urban group had an educational status of high school or above. The majority of mothers were homemakers in both settings. The rural households had larger family sizes with approximately 40% having a family size of five or more compared with 15% in the urban setting.

**Table 1:** Socio-demographic characteristics of the study participants in Ethiopia, 2016.

Characteristics	Descriptions	Urban (n = 85)	Rural (n = 83)	All (n = 168)
**Mothers**				
Age (Years)	Mean (95% CI)	28 (27–29)	34 (32–35)	31 (30–32)
	Median (Min-Max)	28 (19–40)	33 (19–45)	30 (19–45)
Education *n (%)*	No education	5 (6)	40 (48)	45 (27)
	Primary school	28 (33)	34 (41)	62 (37)
	High school	28 (33)	7. (8)	35 (22)
	College and above	24 (28)	2 (2)	26 (16)
Employment *n (%)*	Employed	18 (21)	0 (0)	18 (11)
	Homemaker	59 (69)	48 (58)	107 (64)
	Merchant	8 (9)	5 (6)	13 (8)
	Farmer	0 (0)	30 (36)	30 (18)
Family size n (%)	Three	43 (51)	16 (19)	59 (35)
	Four	28 (33)	16 (19)	44 (26)
	Five	10 (12)	13 (16)	23 (14)
	Six and above	4 (5)	38 (46)	42 (25)
**Children**		Urban (n = 87)	Rural (n = 88)	All (n = 168)
Age (Months)	Mean (95% CI)	6 (5–7)	13 (12–14)	10 (9–11)
	Median (Min-Max)	4 (1–21)	12 (1–24)	9 (1–24)
Sex *n (%)*	Girls	42 (48)	48 (55)	90 (51)
	Boys	45 (58)	40 (46)	85 (49)
Breastfeeding *n (%)*	Yes	82 (94)	85 (97)	167 (95)
Adequately vaccinated for age	Yes	83 (95)	69 (79)	152 (87)

### Detection of potential pathogens in the children and their mothers

The nasopharyngeal samples obtained from the children and the mothers were subjected to bacterial culture for isolation of pneumococci and to multiplex real-time PCR for detection of bacteria and viruses. Pneumococci were isolated by culture from only a few nasopharyngeal samples—in 25/175 (14%) of the children and 13/168 (7.7%) of the mothers. By real-time PCR, *S*. *pneumoniae* were detected in 71% children and 38% of the mothers (Table 2). *H*. *influenzae* was found in 57% of the children and 26% of the mothers, whereas rhinovirus and enterovirus were the most common viruses among both children and their mothers.

**Table 2:** Overall detected bacteria and viruses by multiplex real-time PCR in nasopharyngeal samples obtained from children and their mothers in Ethiopia using the cut-off level Cycle threshold (Ct) <35.

Type of pathogen	Number of positive test results (%)		
	Children (N = 175)	Mothers (N = 168)	Mother-child pair^a^ (N = 164)
**Bacteria**			
*Streptococcus pneumoniae*	124 (71)	60 (38)	42 (26)
*Haemophilus influenzae*	99 (57)	43 (26)	22 (13)
*Bordetella pertussis*	3 (2)	7 (4)	0
*Chlamydia pneumoniae*	1 (0.6)	1 (0.6)	1 (0.6)
*Mycoplasma pneumoniae*	1 (0.6)	1 (0.6)	0
≥1 bacterial species	133 (76)	74 (44)	47 (29)
**Viruses**			
Rhinovirus	75 (43)	31 (18)	12 (7)
Enterovirus	35 (20)	17 (10)	3 (2)
Parainfluenza^b^	11 (6)	3 (2)	0
RSV^c^	7 (4)	7 (4)	1 (1)
Adenovirus	5 (3)	2 (1)	0
Metapneumovirus	5 (3)	0 (0)	0
Bocavirus	1 (0.6)	2 (1.2)	1 (0.6)
Coronavirus^d^	2 (1.1)	0	0
Influenza A	1 (0.6)	1 (0.6)	1 (0.6)
≥1 virus	93 (53)	46 (27)	13 (8)
≥1 bacterial and viral species	78 (45)	27 (16)	6 (4)

^a^ Paired: The same bacterial species or virus type was detected in both the mother and her child.

^b^ Types of parainfluenza viruses detected: type 1 (n = 1) and 3 (n = 10) in the children and type 3 (n = 3) in the mothers.

^c^ RSV: Respiratory syncytial virus.

^d^ Types of coronaviruses detected: Human coronavirus HKU1 (n = 1), and OC43 (n = 2) in the children.

We also analysed the occurrence of the pathogens among the paired children and their mothers and found *S*. *pneumoniae* was present in both the mother and her child in 26% and *H*. *influenzae* in 13% of the mother-child pairs (Table 2).

### Prevalence of detected potential pathogens by PCR in relation to place of residence and fuel type used

As shown in Table 3, a significantly higher proportion of the rural children were positive for *S*. *pneumoniae* (91%) and *H*. *influenzae* (82%) by PCR when compared with the children in the urban setting (51% and 31%, respectively). The prevalence of both bacteria was also significantly higher among mothers from the rural settings compared with the urban group. In addition, the prevalence of both *S*. *pneumoniae* and *H*. *influenzae* was higher among mothers as well as children from households using solid fuels when compared with those using cleaner fuels.

**Table 3:** Prevalence of the most frequently PCR detected bacteria and viruses in nasopharyngeal secretions from Ethiopian children and mothers in relation to place of residence and fuel types used.

Type of pathogen	Children N = 175						Mothers N = 168					
	Rural n (%)	Urban n (%)	*p-value*	Solid fuel n (%)	Cleaner energy n (%)	*p-value*	Rural n (%)	Urban n (%)	*p-value*	Solid fuel n (%)	Cleaner energy n (%)	*p-value*
*S*. *pneumoniae*	80 (91)	44 (51)	**< .001**	98 (81)	26 (48)	**< .001**	40 (48)	20 (24)	**0.001**	48 (41)	12 (23)	**0.022**
*H*. *influenzae*	72 (82)	27 (31)	**< .001**	86 (71)	13 (24)	**< .001**	32 (39)	11 (13)	**< .001**	37 (32)	6 (12)	**0.005**
*B*. *pertussis*	1 (1)	2 (2)	0.55	3 (3)	0 (0)	0.24	0 (0)	7 (8)	**0.014**	5 (4)	2 (4)	0.89
At least one bacterial species	85 (97)	48 (55)	**< .001**	104 (86)	29 (54)	**< .001**	47 (57)	27 (32)	**0.001**	58 (50)	16 (31)	**0.020**
Rhinovirus	44 (50)	31 (36)	0.06	57 (47)	18 (33)	0.09	14 (21)	14 (7)	0.5	22 (19)	9 (17)	0.81
Enterovirus	27 (31)	8 (9)	**< .001**	29 (24)	6 (11)	0.05	8 (10)	9 (11)	0.84	12 (10)	5 (10)	0.89
Parainfluenza	6 (7)	5 (6)	0.74	8 (7)	3 (6)	0.79	0 (0)	3 (4)	0.09	2 (2)	1 (2)	0.93
≥ 1 one virus	53 (60)	40 (46)	0.06	69 (57)	24 (44)	0.12	23 (28)	23 (27)	0.93	32 (28)	14 (27)	0.93
Bacteria and virus	52 (59)	26 (30)	**< .001**	62 (35)	16 (30)	**0.008**	19 (23)	8 (9)	**0.017**	21 (18)	6 (16)	0.28

Among the viruses, only enterovirus showed significantly higher occurrence among rural children compared with the urban groups. Enterovirus and rhinovirus also tended to be more prevalent among children from households using solid fuel compared with those using cleaner energy (*p* = 0.05 and 0.09, respectively) (Table 3).

**Fig 1:**
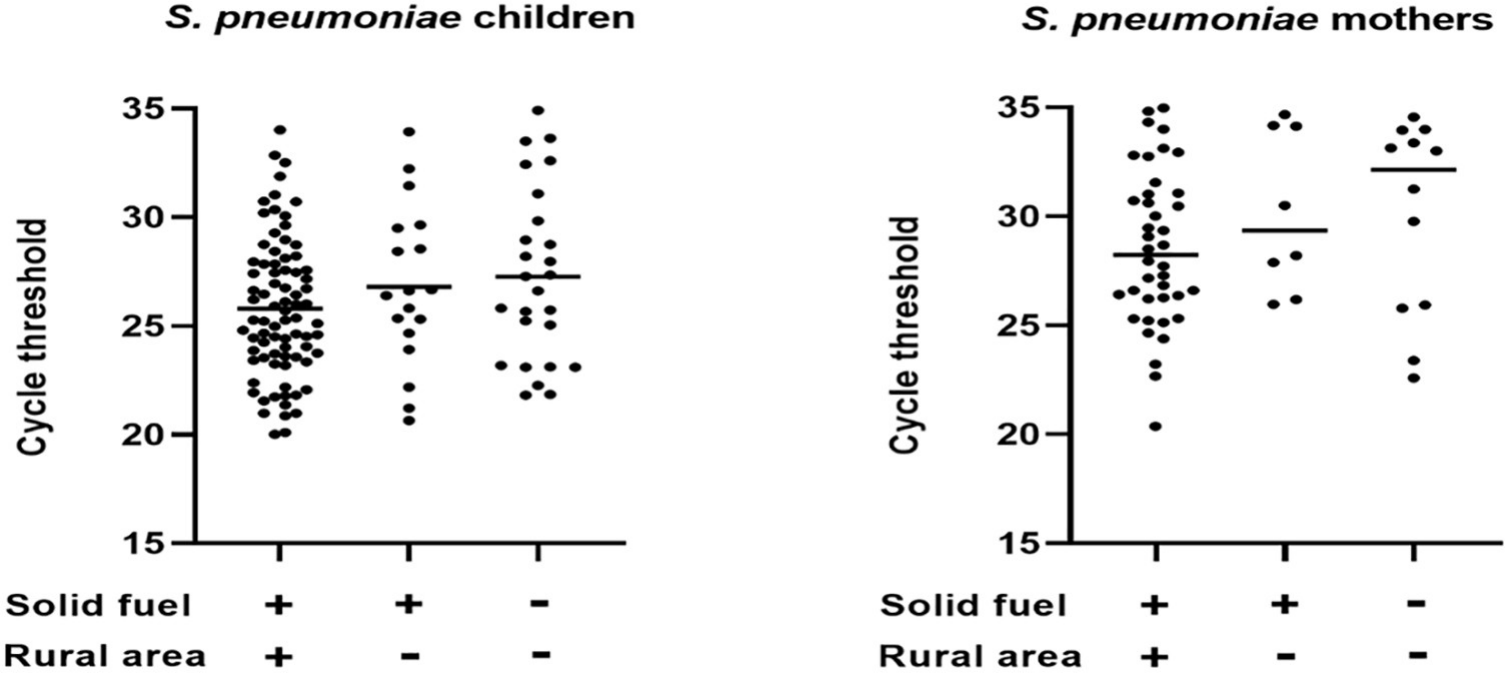
Load of *S*. *pneumoniae* in nasopharynx of children and mothers expressed by Cycle threshold (Ct)-value for detection of the bacteria by PCR in relation to fuel type and place of residence.

Both the children and the mothers from rural areas in households using solid fuel tended to have lower Ct-values of detected pneumococci (i.e. higher bacterial load) compared with cleaner energy users and solid fuel users in the urban setting, but the differences were not significant (Fig 1).

### Factors associated with *S*. *pneumoniae* PCR detection among children

Factors associated with pneumococcal detection were assessed using a multivariable logistic regression model including seven variables, of which only two, fuel type and maternal education, showed association in the final model (Table 4). Rural children, all from households using solid fuel, had more than four times higher odds for detection of *S*. *pneumoniae* compared with those households using cleaner energy in the urban settings. Similarly, children whose mothers did not attend high school or above had four times higher odds of pneumococcal detection. The significant association of other variables in the bivariate model disappeared in the multivariable model.

**Table 4:** Factors associated with detection of *S*. *pneumoniae* by multiplex real-time PCR in nasopharyngeal secretions from the children.

Variables	*S*. *pneumoniae positive (Ct<35)*	Crude Odds Ratio (OR)	Adjusted Odds Ratio (AOR)	*p*-value
	Yes/No (%)			
**Sex of the child**				
Boys	61/24 (72)	1.19 (0.57–2.09)	1.46 (0.66–3.25)	0.35
Girls	63/27 (70)	1	1	
**Fuel type by place of residence**				
Solid fuel rural	80/8 (91)	7.36 (2.85–18.97)	4.39 (1.38–13.92)	**0.012**
Solid fuel urban	18/15 (55)	1.29 (0.542–3.08)	0.86 (0.33–2.26)	0.76
Cleaner energy urban	26/28 (48)	1	1	
**Mothers education**				
Below high school	98/17 (85)	7.53 (3.65–15.57)	4.17 (1.72–10.11)	**0.002**
High school and above	26/34 (43)	1	1	
**Living with other siblings**				
Yes	88/27 (77)	2.17 (1.11–4.26)	1.21(0.52–2.81)	0.66
No	36/24 (60)	1	1	
**Type of ventilation (in the cooking area)**				
Only one door	95/30 (76)	2.29 (1.14–4.56)	1.7 (0.755–3.83)	0.20
Window/permanent opening	29/21 (58)	1	1	
**Presence of at least one type of virus**				
Yes	72/21 (77)	1.98 (1.02–3.83)	1.68 (0.77–3.66)	0.20
No	52/30 (63)	1	1	
**Age of the child**				
Age (months)	(Continuous)	1.09 (1.04–1.16)	1.02 (0.946–1.09)	0.67

### Pneumococcal serotype distribution

**Fig 2:**
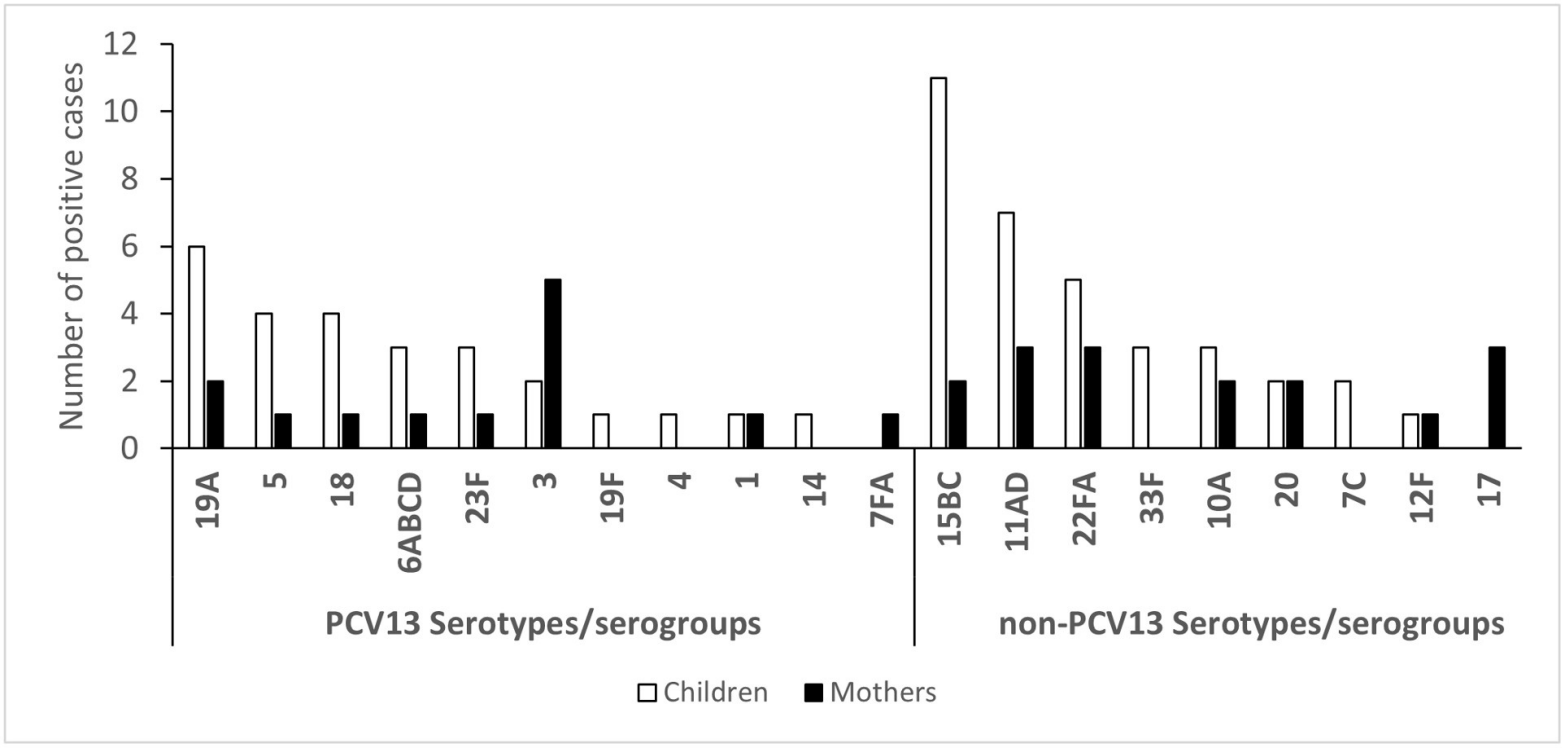
Serotype/serogroups identified directly, by a real-time PCR assay, in nasopharyngeal samples obtained from children and mothers.

From the 124 samples from children and 60 samples from mothers that were positive for pneumococci, 48 (39%) and 26 (43%), respectively, were positive for at least one serotype/serogroup, distributed into 20 different serotypes/serogroups as shown in Fig 2. Two different serotypes were identified in eight children and three serotypes in two children, all these from households using solid fuel, whereas two serotypes were detected in three mothers from households using solid fuel and from households in the rural areas. Thus, a total of 60 pneumococcal serotypes/serogroups were identified in the children of which, 26 (43%) belonged to PCV13, which was similar to the proportion found in the mothers (13/29, 45%) (Fig 2). While 19A was the most prevalent serotype among the children, serotype 3 was the most common serotype found among the mothers. No association between detection of PCV13 serotypes/serogroups and fuel use or living area could be found, although the number of detected serotypes was too low for a thorough statistical analysis. Serotype 19A was also the most prevalent PCV13 serotype detected among those children who had completed PCV13 vaccination. The most common non-PCV13 serogroups in children were 15BC, 11AD and 22FA (Fig 2). However, our PCR panel could not detect any serotype in 76 (61%) of the children nor 34 (57%) of the mothers in samples positive for pneumococci. Since our PCR method was designed to identify all serotypes/serogroups included in PCV13, non-detected serotypes in samples positive for pneumococci were most likely non-PCV13 serotypes.

## Discussion

In this study, we assessed the association of potential respiratory pathogens in nasopharynx with solid fuel use among healthy mothers and their children in urban and rural Ethiopia. We found bacteria more frequently among children and mothers from households using solid fuel compared with those households using cleaner energy. As detected by real-time PCR, *S*. *pneumoniae* was the most prevalent bacterium in nasopharynx of both children and mothers, followed by *H*. *influenzae*. Similar to our findings, studies from Kenya [22], Ghana [7], India [23] and China [24] indicated an association between solid fuel use and higher prevalence of *S*. *pneumoniae* and *H*. *influenzae* in the nasopharynx using either culture or PCR. However, other studies reported no association between fuel use and carriage of these bacteria [25,26] nor with place of residence [27]. In this study, there was no significant association between the use of fuel and detection of viruses. However, there was a significantly higher detection of enterovirus among children in rural areas compared with those from urban areas.

As expected the proportion of detected *S*. *pneumoniae* was higher among the children compared with the mothers. This is consistent with previous reports indicating that the rate of *S*. *pneumoniae* carriage becomes lower as the age increases [28,29]. A longitudinal study from Thailand also showed that mothers acquired pneumococci less frequently and continued to carry them for shorter periods compared with children [30]. This could be linked with the duration of carriage which is longer for children than for adults [31]. Among the children we could not show any association between age and pneumococcal detection, but the proportion of detected *S*. *pneumoniae* were higher among the rural when compared with urban children.

Vaccination against both pneumococci (PCV13) and *H*. *influenzae* type b (included in the Pentavalent vaccine) are given simultaneously to Ethiopian infants at age of 6, 10 and 14 weeks [19], and 89% of the urban and 79% of the rural participating children were hence adequately protected by this combination vaccines for their age. Our method could not distinguish between encapsulated *H*. *influenzae*, including the highly virulent type b, and non-encapsulated (non-typeable) *H*. *influenzae*. This might explain why 57% of the children had *H*. *influenzae* detected in their samples despite completed Hib vaccination. We found that 26 (43%) of the detected serotypes/serogroups of *S*. *pneumoniae* were vaccine serotypes and 20 of these were detected from children who had completed the three-dose schedule for PCV13. The pneumococcal serotype 19A, which is included in PCV13, was detected in six children, thus being more prevalent than other vaccine serotypes This was similar to the findings reported in previous studies in Ethiopia [32,33] and other countries [34–36]. Nevertheless, non-PCV13 serotypes/serogroups 15BC and 11AD were the most frequently detected serotypes among the children similar to other studies in Tanzania and the Democratic Republic of the Congo [37,38]. However, since our PCR panel was designed to mainly detect PCV13 serotypes/serogroups, and no serotype could be detected in 60% of the pneumococcal positive samples, our results indicate the vast majority of carried pneumococci to be non-PCV13 serotypes in both children and the mothers.

The odds of detecting *S*. *pneumoniae* was more than four times higher for children from households using solid fuel compared with cleaner energy users. This is in line with the evidence from a prospective, observational study from Malawi, which reported that one unit increase in decile of PM2.5 exposure increased the risk of S. *pneumoniae* carriage by 10% among infants [8]. This is also consistent with previous studies conducted on children in Tanzania [37] and Indonesia [26] and on mothers and infants in South Africa [5]. In addition, there have also been multiple studies consistently reporting the association between solid fuels and acute lower respiratory infections in children [15,39,40]. In contrast, a systematic review reported no associations between *S*. *pneumoniae* carriage or acute lower respiratory infections and solid fuel use among adults [41].

Our multivariable analysis also showed that detection of *S*. *pneumoniae* among the children was affected by the educational levels of their mothers, as previously reported by other authors [26]. We found that children whose mothers had not attended school or who had an educational status below high school had four times higher odds of having positive results for pneumococci compared with those children whose mothers had attended high school or above. A review of studies in developing countries reported that those mothers who had attended schooling to secondary level and/or above showed higher health care utilization and lower mortality due to pneumonia among their children. This was as a result of an improved understanding of and diseases causation [42]. However, research from Niger on a similar age group reported that pneumococcal carriage was not associated with maternal education [43]. The differences might be due to the study settings as they included hospitalised children. In this study, low maternal education might be associated with low socio-economic standard with use of solid fuel on open fire and crowding in the houses, although we found no association between pneumococcal detection and family size.

Strengths of this study included involvement of both mothers and their children from rural and urban settings in the country and an assessment of the association of potential pathogens with fuel types used, not previously undertaken in Ethiopia. Our method was to detect a broad panel of bacteria and viruses, most of which have been known to cause severe infection in the respiratory tract.

However, our findings have the following limitations. We did not measure the level of household air pollution and could not include factors such as child birth weight and monthly income (reported in other studies) owing to a lack of data, especially in the rural settings. Some of the rural mothers did not have a vaccination card; thus, we had to rely on self-reported information. The overall vaccination coverage in the villages was reported to be higher than found in this study. Not being able to identify all serotypes in the pneumococcal positive samples by the PCR method is another limitation affecting the possibility to relate pneumococcal serotypes to socio-demographic factors. Pneumococci could be isolated by culture from only a few nasopharyngeal samples. We suggest this may be linked to methodological shortcomings. These have led to the activation of the pneumococcal autolysin enzyme and death of the bacteria. Thus, no analysis of pneumococcal carriage as detected by culture and its relation to household air pollution or other factors could be performed. This also limited analysis of bacteria strains carried both by the mother and the child.

## Conclusions

In conclusion, our study has found high rates of potential respiratory pathogens, including *S*. *pneumoniae* and *H*. *influenzae*, in the upper respiratory tract of children and their mothers. Our results also indicated that children from households using solid fuel and with a maternal education status below high school had higher odds of nasopharyngeal detection of *S*. *pneumoniae*. On the basis of our results, we propose that interventions should target the provision of cleaner energy sources for the communities and promoting education of women below child-bearing age. Future longitudinal studies at community level could be of value to measure the causality along age difference.
